# Impaired Mitochondrial DNA Copy Number in Visceral Adipose Tissue of Insulin-Resistant Individuals: Implications for Metabolic Dysregulation

**DOI:** 10.3390/ijms26157398

**Published:** 2025-07-31

**Authors:** Monika Ołdakowska, Aneta Cierzniak, Tomasz Jurek, Małgorzata Małodobra-Mazur

**Affiliations:** 1 Department of Forensic Medicine, Division of Molecular Techniques, Wroclaw Medical University, Sklodowskiej-Curie 52, 51-367 Wroclaw, Poland; monika.oldakowska@umw.edu.pl (M.O.);; 2 Genomtec S.A., Ul. Bierutowska 57-59, 51-317 Wroclaw, Poland; 3 Department of Forensic Medicine, Wroclaw Medical University, Jana Mikulicza-Radeckiego 4, 50-345 Wroclaw, Poland; tomasz.jurek@umw.edu.pl

**Keywords:** insulin resistance, mitochondrial DNA (mtDNA), visceral adipose tissue, subcutaneous adipose tissue

## Abstract

Insulin resistance is a fundamental pathophysiological mechanism contributing to the development of type 2 diabetes and metabolic syndrome. Recently, attention has focused on mitochondria’s role in glucose and lipid metabolism. Mitochondrial dysfunction is strongly associated with impaired energy metabolism and elevated oxidative stress. We investigated the mitochondrial DNA (mtDNA) copy number in subcutaneous adipose tissue (SAT) and visceral adipose tissue (VAT) in insulin-sensitive (IS) and insulin-resistant (IR) individuals. Twenty-seven paired adipose tissue biopsies were obtained during elective abdominal surgery. DNA and RNA were extracted, and mtDNA copy number was quantified using Real-Time PCR. We found that mtDNA content in VAT was approximately two-fold lower than in SAT. Furthermore, in IR individuals, mtDNA copy number was significantly reduced in both SAT and VAT compared to IS subjects. A strong positive correlation was observed between mtDNA content in VAT and body mass index (BMI), and a negative correlation was found with the QUICKI index. Additionally, mtDNA copy number in VAT positively correlated with the expression of several genes involved in insulin signalling, lipid metabolism, and other metabolic pathways. These findings underscore the central role of mitochondrial function in VAT in the context of metabolic disorders and suggest that targeting mitochondrial regulation in this tissue may represent a promising therapeutic approach.

## 1. Introduction

Insulin resistance is a fundamental pathophysiological mechanism contributing to the development of type 2 diabetes (T2D) and metabolic syndrome [1]. In recent years, growing attention has been directed towards the role of mitochondria in the regulation of glucose and lipid metabolism, and their dysfunction is increasingly indicated as one of the key factors in the development of insulin resistance [2]. Mitochondria play a central role in cellular energy homeostasis, participating in the electron transport chain, resulting in ATP production, while generating reactive oxygen species (ROS) [3]. In healthy individuals, elevated insulin levels stimulate increased mitochondrial ATP production in muscles, enhance protein synthesis, and boost the enzymatic activity of cytochrome C oxidase and citrate synthase. Conversely, insulin-resistant individuals exhibit a diminished mitochondrial response to insulin [4,5]. Similarly, individuals with type 2 diabetes demonstrate reduced insulin-stimulated rates of mitochondrial ATP production in muscles. This impaired mitochondrial response to insulin has been demonstrated using magnetic resonance spectroscopy (MRS), where ATP synthesis rates failed to increase under hyperinsulinemic-euglycemic clamp conditions in T2DM patients. Stump et al. found that while insulin-stimulated ATP production increased by over 30% in healthy individuals, subjects with type 2 diabetes exhibited a blunted or absent response (average increase <10%) despite comparable insulin levels [6].

Mitochondria contain their own genome, designated as mitochondrial DNA (mtDNA). In humans, mtDNA is a double-helix, circular, closed molecule, comprising 16,569 base pairs. It exists in numerous copies within each mitochondrion, while each cell contains various numbers of mitochondria that mainly depend on the cell type and physiological conditions [7]. Assessment of mtDNA copy number enables the identification of mitochondrial dysfunction in large-scale research or clinical groups. This marker reflects the ratio between mtDNA and nuclear DNA within the cell and is associated with mitochondrial enzyme activity and ATP production, which are the key indicators of mitochondrial health [8]. Furthermore, variation in mitochondrial DNA copy number has been identified as a potential marker of metabolic disorders [9]. Reduction in mtDNA copy number in insulin-sensitive tissues, such as skeletal muscle and adipose tissue, is associated with impaired mitochondrial function and an elevated risk of developing type 2 diabetes [9].

Mitochondrial dysfunction is strongly associated with impaired energy metabolism and increased oxidative stress, both of which can contribute to impaired insulin signalling [10]. Studies indicate that individuals with insulin resistance exhibit a reduced number of mitochondria and diminished mitochondrial function in muscle tissue, leading to impaired regulation of carbohydrate and lipid metabolism [10,11]. Moreover, abnormalities in mitochondrial biogenesis and decreased oxidative activity contribute to lipid accumulation in insulin-dependent tissues, further exacerbating insulin resistance [12]. In addition, disturbances in mitochondrial dynamics, including the process of fusion and fission, also have a significant impact on cellular energy homeostasis. Abnormal mitochondrial fragmentation, often observed in insulin-resistant states, results in reduced efficiency of the respiratory chain and increased production of reactive oxygen species (ROS). These ROS, in turn, induce oxidative stress that can disrupt insulin signalling pathways by interfering with the phosphorylation of key proteins such as IRS-1 and AKT [13,14].

Many studies have focused on mitochondrial function in skeletal muscle and blood cells. The amount of peripheral blood mtDNA has been shown to correlate with insulin sensitivity, which suggests that this parameter may be an indicator of insulin sensitivity [15]. Mitochondrial dysfunction in skeletal muscle cells is closely associated with insulin resistance and type 2 diabetes [2,16]. In addition, impaired mitochondrial function in muscles has been observed in both young and older individuals with type 2 diabetes [17]. In adipose tissue, mitochondria are primarily responsible for cellular energy production. Moreover, they play a crucial role in adipocyte biology, including adipogenesis, lipid metabolism, and thermogenesis [18,19]. Proper mitochondrial function in adipose tissue is essential for maintaining energy homeostasis and ensuring the effective operation of insulin signalling pathways [20,21]. Disturbances in mitochondrial function, such as a decrease in their number, reduced enzymatic activity, or increased oxidative stress, are increasingly associated with the development of insulin resistance [21,22]. In white adipose tissue, mitochondrial dysfunction has been shown to be associated with abnormal lipid metabolism, leading to the accumulation of sphingolipids, mainly ceramides, which interfere with insulin signalling [20,23]. Additionally, oxidative stress and mitochondrial dysfunction can exacerbate inflammation, further aggravating insulin resistance [11,22]. As a result, cells become less responsive to insulin, which is a significant risk factor in the pathogenesis of type 2 diabetes and other metabolic disorders [21,23].

Based on literature reports, it is known that there are differences between subcutaneous adipose tissue (SAT) and visceral adipose tissue (VAT), which are reflected in their distinct anatomical location, cellular composition, and hormonal function [24]. VAT has been associated with adverse metabolic outcomes, including insulin resistance, type 2 diabetes, and cardiovascular disease, due to its higher lipolytic activity, pro-inflammatory profile, and proximity to the portal vein circulation, which facilitates the delivery of free fatty acids and cytokines to the liver [24,25]. In contrast, SAT is considered to be less harmful and metabolically active and may even exert protective effects. Based on this, VAT is considered to be a fat storage site contributing to systemic metabolic dysfunction, especially insulin resistance [26].

Therefore, in the present study, we aim to investigate the differences in mitochondrial DNA mtDNA copy number between SAT and VAT in insulin-sensitive and insulin-resistant individuals to capture the specific mitochondrial alterations between these two fat depots that may underlie the differential involvement of these tissues in metabolic diseases.

## 2. Results

### 2.1. Characteristic of Study Cohort

The study cohort was divided into insulin-sensitive (IS) and insulin-resistant (IR) individuals based on HOMA-IR and QUICKI ratios. Individuals possessing HOMA > 2.5 and QUICKI < 0.321 were classified as insulin resistant. Investigated groups did not differ significantly in terms of glucose level, which was only slightly higher in the IR group. On the other hand, insulin levels, as well as HOMA-IR, were significantly increased in IR patients, with significantly lower QUICKI values. IR patients were also characterized by significantly higher triglyceride levels and lower HDL levels. The characterization of the enrolled subjects, with division into studied groups, is presented in Table 1.

### 2.2. MtDNA Content in Adipose Tissue Biopsies in IR and Is Subjects

**Figure 1:**
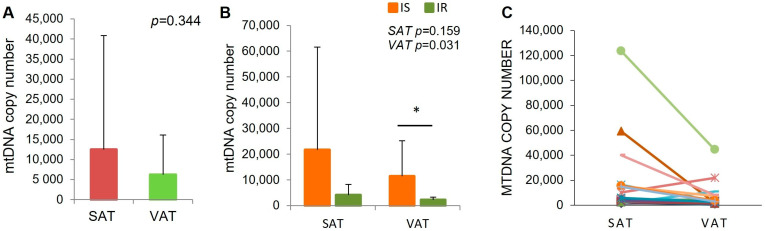
mtDNA copy number assessed in SAT and VAT (**A**) and with subdivision of enrolled subjects into IS and IR (**B**), paired analysis of mtDNA copy number between SAT and VAT measurements. Colors correspond to individual participants (**C**). N-27 (IS-13; IR-14), * *p*-statistically significance < 0.05.

Higher mtDNA content was observed in subcutaneous adipose tissue compared to visceral adipose tissue, although without statistical significance. The mean mtDNA content in VAT was about two times lower compared to SAT (Figure 1A).

The mtDNA content in both SAT and VAT was lower in IR individuals, compared to IS subjects, which might suggest mitochondrial depletion in insulin resistance. However, the difference was statistically significant only for VAT (*p* = 0.0317, Figure 1B, Table 2).

Additionally, paired analyses showed lower mtDNA content in VAT in almost all analyzed individuals (Figure 1C).

**Figure 2:**
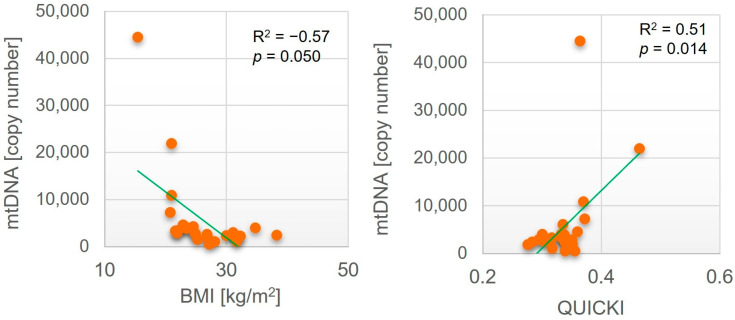
Correlation between mtDNA copy number and BMI and QUICKI in visceral adipose tissue of enrolled subjects. N-27 (IS-13; IR-14).

In order to fully evaluate the relationship between mtDNA copy number and insulin signaling and sensitivity to insulin, we searched for the relationship between mtDNA content and biochemical parameters. mtDNA copy number in SAT negatively correlated with age (*p* = 0.002). No further statistically significant correlation was observed. On the other hand, the mtDNA copy number in VAT negatively correlated with BMI (R^2^ = −0.57, *p* = 0.050) within enrolled individuals and positively with QUICKI (R2 = 0.51, *p* = 0.014, Table 3, Figure 2). The strong positive correlation with QUICKI might suggest a relationship with insulin resistance. It is worth mentioning that a negative correlation between insulin level (R2 = −0.31) and HOMA (R2 = −0.31) was observed, although without statistical significance (Table 3). Additionally, negative correlation with BMI also links obesity with a decrease in mtDNA copy number, thus with depletion of mitochondrial content in VAT in obese or overweight individuals

### 2.3. Analysis of Gene Expression and Correlation with mtDNA Content in SAT and VAT

**Figure 3:**
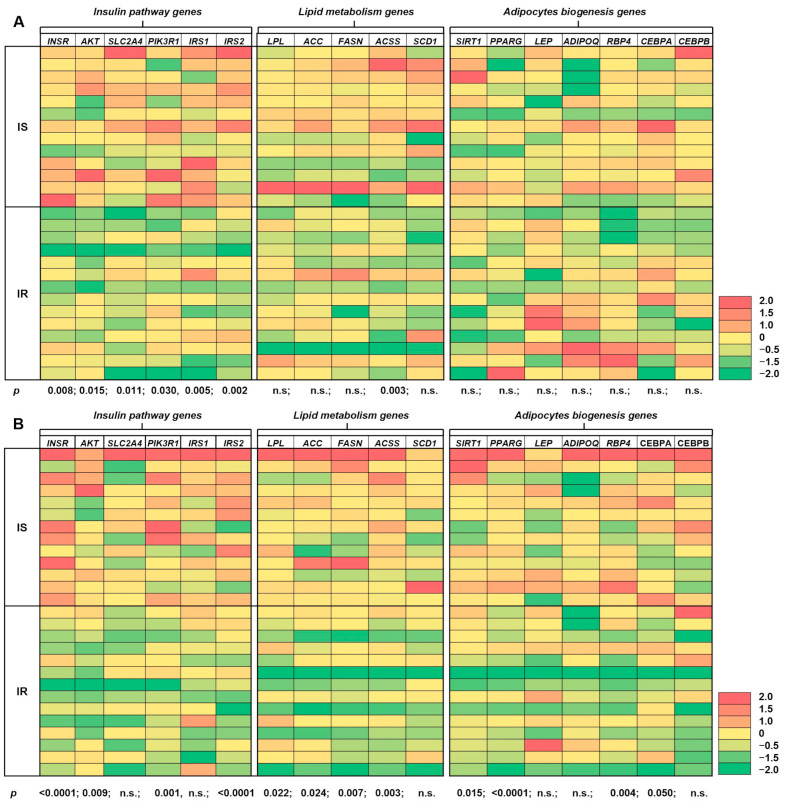
Expression of insulin signaling genes, lipid metabolism genes, as well as other genes regulating mostly adipogenesis and other aspects of adipocyte metabolism in SAT (**A**) and VAT (**B**) in enrolled subjects. The expression level is represented as an increase in relation to the mean (0 represents the mean expression for the individual gene in IS subjects). n.s.—statistically not significant. N-27 (IS-13; IR-14).

To further evaluate the relationship between mtDNA copy number and sensitivity to insulin, we measured expression of numerous genes in both SAT and VAT, mostly related to the insulin signaling pathway, lipid metabolism, inflammatory genes, and other genes involved in adipogenesis and regulation of basic adipocyte metabolism. The significant decrease in expression in all of the investigated genes belonging to insulin signaling pathway, including *INSR* (*p* = 0.008), AKT (*p* = 0.015), *SLC2A4* (*p* = 0.011), *PIK3R1* (*p* = 0.030), *IRS1* (*p* = 0.005) and *IRS2* (*p* = 0.002) was detected in SAT collected from IR subject comparing to IS individuals. No further statistically significant divergences in gene expression were detected in SAT (Figure 3A). VAT biopsies collected from IR subjects were characterized by decrease expression of numerous genes belong to insulin signaling pathway (*INSR p* < 0.0001, *AKT p* = 0.009, *PIK3R1 p* = 0.001, *IRS2 p* < 0.0001), lipid metabolism (*LPL p* = 0.022, *ACC p* = 0.024, *FASN p* = 0.007, *ACSS p* = 0.003) and others (*SIRT1 p* = 0.015, *PPARG p* < 0.0001, *RBP4 p* = 0.004, *CEBPA p* = 0.050) comparing to subjects with proper insulin sensitivity (Figure 3B).

**Figure 4:**
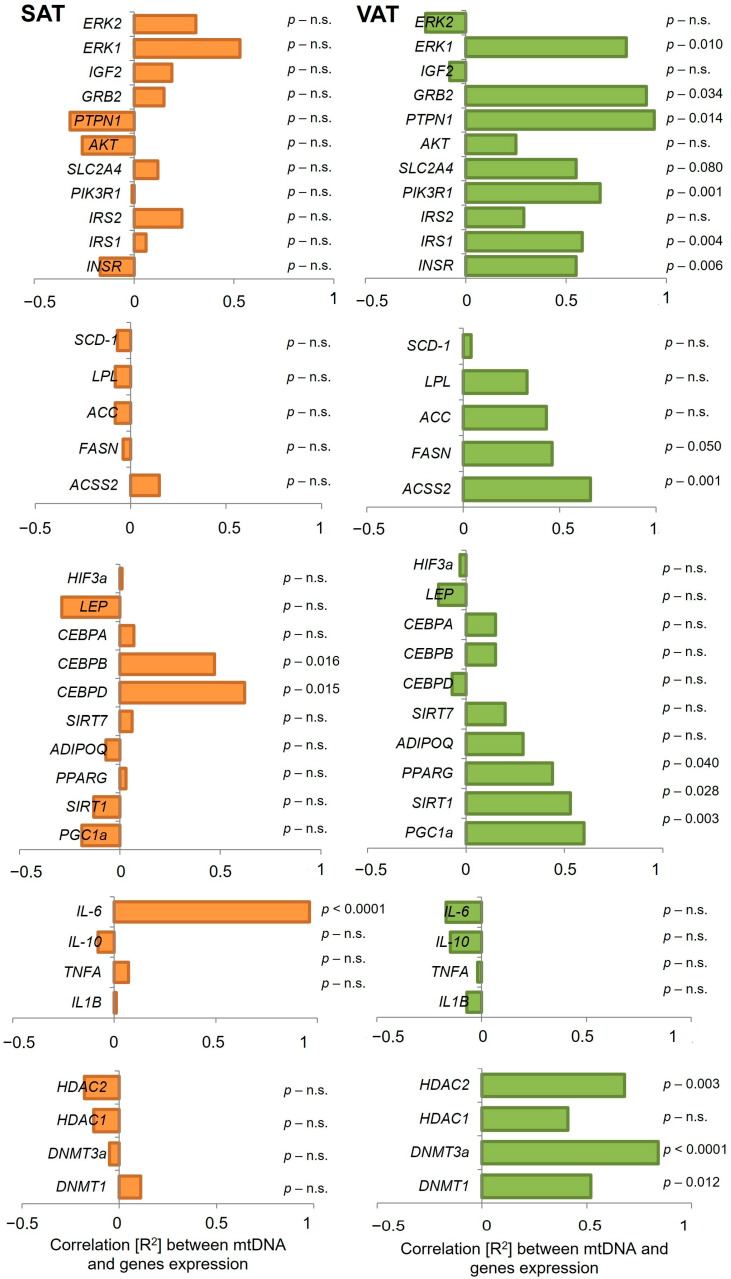
The correlation between mtDNA copy number and expression of insulin pathway genes, lipid metabolism genes, adipocyte metabolism genes, inflammatory genes, and epigenetic regulatory genes in SAT and VAT of enrolled subjects. N-27 (IS-13; IR-14), n.s.–statistically not significant.

To further evaluate the relationship between mtDNA copy number and sensitivity to insulin, we evaluated the correlation between the expression of numerous genes with mtDNA copy number in SAT and VAT within enrolled subjects. We have detected a significant correlation between the expression of *IL-6*, *CEBPB*, and *CEBPD* genes and mtDNA copy number in subcutaneous adipose tissue (Figure 4). An interesting observation was noticed between the expression of the *IL-6* gene and mtDNA content in SAT, where a strong positive correlation (R^2^ = 0.96, *p* < 0.0001) was shown. Furthermore, mtDNA copy number positively correlated with *CEBPB* and *CEBPD* genes (R^2^ = 0.47, *p* = 0.016 and R^2^ = 0.62, *p* = 0.015, respectively). No further relationship was observed.

In contrast, in VAT the expression of most of investigated genes showed strong positive correlation with mtDNA copy number, including insulin pathway genes: *INSR* (R2 = 0.55, *p* = 0.006), *IRS1* (R2 = 0.58, *p* = 0.004), *PIK3R1* (R2 = 0.67, *p* = 0.001), PTPN1 (R2 = 0.94, *p* = 0.014) and lipid metabolism genes: *ACSS2* (R2 = 0.66, *p* = 0.001), *FASN* (R2 = 0.46, *p* = 0.033) and *ACC* (R2 = 0.43, *p* = 0.050, Figure 4). No statistically significant correlation was observed for inflammatory genes; on the other hand, mtDNA copy number in VAT positively correlated with expression of PPARG (R2 = 0.44, *p* = 0.040), *SIRT1* (R2 = 0.53, *p* = 0.028), and *PGC1a* (R2 = 0.60, *p* = 0.001, Figure 4) of enrolled subjects.

We have also noticed an interesting relationship between mtDNA copy number in VAT with epigenetic regulatory genes, among others *HDAC2* (R2 = 0.68, *p* = 0.003), *DNMT3a* (R2 = 0.84, *p* < 0.0001), and *DNMT1* (R2 = 0.52, *p* = 0.012).

We also examined the correlations within the IS and IR groups separately and observed a similar pattern of association. Only a few genes expressed in SAT, namely *CEBPB*, *CEBPD*, and *IL-6*, showed a correlation with mtDNA copy number. However, *IL-6* exhibited a significant correlation only in IS individuals, with no correlation observed in the IR group (Table 4).

Most of the evaluated genes expressed in VAT were correlated with mtDNA copy number when analyzed separately by group. Notably, the majority of statistically significant correlations were found in IR patients. These included genes involved in the insulin signaling pathway, lipid metabolism, and adipokine synthesis (Table 4). In contrast, while correlations were also detected in IS individuals, they were weaker and did not reach statistical significance.

## 3. Discussion

In the present article, we demonstrated how the mtDNA copy number varies in the two most abundant adipose tissue compartments, that is, subcutaneous adipose tissue (SAT) and visceral adipose tissue (VAT) in insulin-sensitive and insulin-resistant individuals. Based on the obtained results, we observed that the number of mtDNA copies in SAT was almost twice as high as compared to VAT, suggesting that visceral fat showed lower mitochondrial activity compared to subcutaneous fat. Our results are consistent with the findings of Kraunsøe et al., who demonstrated significantly lower mitochondrial respiration in VAT, as indicated by mtDNA copy number [27]. Interestingly, despite this observation, the authors showed that visceral adipose tissue is characterized by a significantly higher number of mitochondria per milligram of tissue, along with greater coupled and uncoupled respiration than subcutaneous adipose tissue. They concluded that VAT is bioenergetically more active and more responsive to mitochondrial substrate supply than SAT [27]. Therefore, it can be assumed that although VAT contains more mitochondria per unit mass, it exhibits higher oxidative activity per unit mass of tissue, yet potentially lower mitochondrial efficiency compared to SAT. These differences may result from distinct metabolic functions and adaptations between the two types of adipose tissue. Despite the difference in mtDNA copy number between SAT and VAT, we showed lower mtDNA copy number in IR individuals in both fat depots; moreover, in visceral adipose tissue, insulin resistance has been associated with a significant reduction in mtDNA copy number. This is most likely due to the fact that insulin resistance is a pathological state in which impaired ATP production in mitochondria leads to a failure of pancreatic β cells to secrete a sufficient amount of insulin. Our findings support the hypothesis that mitochondrial damage is one of the key contributing factors to IR development [28].

The observed decrease in mtDNA copy number in visceral adipose tissue (VAT) of insulin-resistant individuals may result from several interrelated mechanisms that impair mitochondrial genome stability and turnover. One of the most important factors is mitophagy, the selective degradation of damaged mitochondria via autophagy, which is crucial for maintaining mitochondrial quality control and energy efficiency [19]. This process is crucial for the quality of mitochondria and depends on signaling functions (e.g., PINK1/Parkin pathway) and on the ULK1 kinase activated by AMPK or inhibited by mTORC [19]. In healthy adipocytes, mitophagy prevents the accumulation of dysfunctional organelles and limits excess ROS production. However, in obesity and insulin-resistant states, mitophagy becomes dysregulated, partly due to nutrient overload and chronic mTORC1 activation, which inhibit the ULK1 complex responsible for autophagy initiation [19,29]. Impaired mitophagy may reduce mitochondrial turnover and may lead to increased accumulation of damaged mitochondria, which in turn contributes to metabolic dysfunction in adipose tissue [19]. Experiments in yeast have shown that cells lacking mitophagy genes accumulate excess mitochondria, resulting in ROS overproduction and secondary mtDNA mutations. In wild-type mitophagy, this phenomenon is prevented by reducing the number of mitochondria to a bare minimum and suppressing ROS generation [30]. Thus, excessive mitochondrial damage in combination with insufficient mitophagic clearance may reduce mtDNA content through increased degradation or failed replication of damaged genomes. In addition to impaired mitophagy, direct disruption of mtDNA replication and maintenance mechanisms may contribute to the reduction in mtDNA copy number. The replication of mtDNA depends on DNA polymerase γ (POLG) and mitochondrial transcription factor A (TFAM), both essential for mtDNA stability [31]. Mutations or dysregulation of these components have been shown to limit mtDNA replication and lead to depletion of mtDNA in metabolically active tissues [32,33,34,35]. The non-coding D-loop region of the mitochondrial genome is particularly important, as it contains the origin of replication and is susceptible to mutations under oxidative stress conditions [36]. Notably, oxidative damage is a major contributor to mtDNA instability in insulin-resistant VAT, where excessive ROS production leads to the formation of base modifications such as 8-oxo-dG and strand breaks [7,37]. These lesions, in the context of limited mitochondrial DNA repair capacity, result in degradation of mtDNA rather than its preservation [37]. Thus, a reduced mtDNA copy number may reflect not only mitochondrial loss but also impaired copying or increased catabolism of the mitochondrial genome [38,39]

We observed the negative correlation between BMI and the number of mtDNA copies in visceral adipose tissue, suggesting that individuals with a higher BMI have fewer mtDNA copies (by this means fewer mitochondria) in adipose tissue. This finding may indicate mitochondrial dysfunction in the context of obesity. In obese individuals, mitochondria within adipose tissue may be reduced in number or exhibit impaired function, a condition associated with metabolic disorders and insulin resistance. Similarly, other studies have also reported a negative correlation between mtDNA copy number and BMI, supporting the fact that obesity is linked to a decrease in mtDNA copy number in visceral fat tissue [7,40]. This reduction may impair the tissues’ ability to metabolize fat and glucose effectively, potentially leading to further fat accumulation and contributing to metabolic diseases such as insulin resistance and diabetes [40]. Supporting the above finding, the positive correlation between the QUICKI Index and mtDNA copy number may indicate that, in the presence of higher insulin resistance, the number of mitochondria in adipose tissue decreases, which remains consistent with the finding of Sergi et al. In their reports, it was demonstrated that mitochondrial dysfunction in adipose tissue is associated with the pathogenesis of insulin resistance, a hallmark of type 2 diabetes. The authors proposed that improving mitochondrial function may offer a promising therapeutic strategy to enhance insulin sensitivity [41]. The observed decrease in mitochondria in VAT that correlated with insulin resistance clearly indicates a relationship between the two. Taken together, these correlations highlight a complex interplay between energy metabolism, mitochondrial function, obesity, and insulin resistance in adipose tissue, in particular in VAT [21,42,43].

Reduced expression of insulin signalling pathway genes in patients with insulin resistance (*INSR*, *AKT*, *PIK3R1*, *IRS2*) indicates a diminished insulin response in both VAT and SAT. These genes play a critical role in insulin signalling, which regulates glucose uptake and lipid metabolism. Their downregulation suggests impaired insulin receptor activation and disrupted downstream signalling, resulting in reduced glucose uptake, altered lipolysis, and compromised lipid storage capacity in adipocytes [44,45]. Furthermore, chronic suppression of insulin-responsive gene expression has been associated with increased inflammation and oxidative stress in adipose tissue, which may further exacerbate insulin resistance through feedback inhibition of insulin signalling components [46].

Reduced expression of lipid metabolism genes (*LPL*, *ACC*, *FASN*, *ACSS*) in patients with insulin resistance in VAT indicates significant disturbances in lipid handling. The downregulation of these genes may result in inefficient lipid clearance, elevated circulating fatty acid levels, and altered lipid partitioning, all of which contribute to adipocyte dysfunction and increased insulin resistance [47,48]. The absence of significant differences in lipid gene expression and metabolism in SAT may be attributed to the fact that VAT is more metabolically active and secretes greater amounts of adipokines, which can influence insulin resistance [49]. VAT is also more sensitive to lipolysis, which leads to the greater release of free fatty acids (FFAs) into the bloodstream [50]. Excessive accumulation of VAT is strongly associated with insulin resistance, type 2 diabetes, and cardiovascular disease. In present studies, we have demonstrated that VAT exhibits distinct gene expression patterns compared to SAT, which contributes to differences in metabolic function and their respective roles in insulin resistance [51]. Notably, VAT exhibits increased expression of genes associated with inflammation, oxidative stress, and lipid metabolism disorders [52].

We also observed a reduced expression of key genes crucial for adipose tissue biogenesis and adipogenesis in VAT in insulin-resistant compared to insulin-sensitive individuals, including *SIRT1* (sirtuin 1), *PPARG* (peroxisome proliferator-activated receptor gamma), *RBP4* (retinol binding protein 4), and *CEBPA* (C/EBP enhancer binding protein alpha). The downregulation of these genes indicates that adipocyte dysfunction may contribute to insulin resistance and other metabolic disorders. A significant reduction in *SIRT1* expression in VAT of insulin-resistant patients indicates disturbances in the regulation of energy metabolism and impaired ability to maintain insulin sensitivity [53]. Similarly, a reduction in *PPARG* expression indicates insulin resistance, as PPARG is the main regulator of sensitivity to insulin, but also suggests disturbances in lipid homeostasis, which may lead to lipotoxicity [54]. Additionally, reduced *CEBPA* expression, which plays a pivotal role in adipocyte differentiation, intensifies metabolic disorders and leads to abnormal lipid storage, further contributing to the development of insulin resistance [55]. In contrast, no analogous differences in the expression of the discussed genes were observed in SAT between the IR and the IS individuals. This observation highlights the distinct metabolic function between SAT and VAT, with VAT being more strongly associated with metabolic disorders and showing greater abnormalities in gene expression [24].

Our analysis of the correlation between mtDNA copy number and insulin signalling pathway gene expression in VAT and SAT revealed that VAT has a greater impact on metabolism and is more actively involved in metabolic disorders. This is reflected in the higher expression of genes related to insulin signalling. We observed a strong positive correlation between mtDNA copy number and expression of *ERK1, GRB2, PTPN1, SLC2A4, PIK3R1, IRS1,* and *INSR*, the correlation was observed inclusively in VAT. Subcutaneous adipose tissue is less metabolically active and primarily functions in long-term energy storage rather than in intensive participation in the regulation of metabolism [24]. In contrast, VAT is metabolically dynamic and plays a central role in regulating metabolism, particularly in the context of insulin resistance and related metabolic disorders [56,57,58]. The elevated expression of insulin signalling pathway genes in VAT may reflect its more substantial role in insulin responsiveness and glucose metabolism.

Similarly, the strong positive correlation between mtDNA copy number and the expression of lipid metabolic genes such as *ACC, FASN*, and *ACSS2* observed only in VAT and not in SAT, suggests a specific relationship between mitochondrial activity and lipid metabolic processes in VAT [59]. This implies that in VAT, mitochondrial function is more directly involved in the regulation of lipid metabolism pathways. Mitochondria in VAT may play a key role in regulating energy and lipid metabolism. In this tissue, mitochondria are more active, which may lead to a higher mtDNA copy number and thus a greater effect on the expression of genes related to lipid metabolism. In contrast, the absence of such correlation in SAT may suggest that lipid metabolism in SAT may be less dependent on mitochondrial function or may be governed by different regulatory mechanisms [60,61]. These findings underscore fundamental differences in mitochondrial function and lipid metabolism between the two adipose tissue types.

The positive correlation between mitochondrial DNA (mtDNA) copy number and expression of genes regulating adipocyte metabolism, such as *PPARG, SIRT1, and PGC-1α*, in adipose tissue suggests that increased mitochondrial content may support and enhance metabolic functions in fat cells [62]. These genes play central roles in metabolism regulation, adaptation to energy stress, and maintenance of energy homeostasis in adipocytes, with mitochondria serving as a key organelle for energy production [63,64,65]. Mitochondria are responsible for ATP production via the respiratory chain and play a critical role in energy storage and release in adipocytes, particularly through processes such as lipolysis. Increased number and enhanced function of mitochondria may enable a more efficient response to energy demands in adipose tissue [66]. In brown and beige adipose tissue, mitochondria are especially important for thermogenesis, the process of producing heat, which may be linked to the regulation of genes such as PGC-1α. This gene supports mitochondrial biogenesis and metabolic regulation, so its increased expression may lead to an increase in the number of mitochondria in fat cells and thereby boost metabolic functions in adipocytes [67].

PPARG regulates adipocyte differentiation and lipid storage, but also contributes to the metabolic activity of these cells, which may be further supported by higher mitochondrial counts [68]. Increased mtDNA copy numbers may support PPARG metabolic pathways by providing greater mitochondrial capacity for lipid handling and energy production [69]. SIRT1 is closely associated with key metabolic functions, including mitochondrial activity and energy processes in adipocytes, helping to increase their ability to burn fat. It also enhances fatty acid oxidation, reduces inflammation, and improves insulin sensitivity in adipose tissue, linking mitochondrial activity to broader systemic energy regulation [41]. The observed correlation between mtDNA copy number and the expression of *PPARG, SIRT1*, and *PGC-1α* exclusively in VAT suggests a coordinated regulation of mitochondrial biogenesis and lipid metabolism in this depot. This may reflect a higher metabolic demand in VAT, necessitating increased mitochondrial activity to support its function [70].

We demonstrated a strong positive correlation between expression of the IL-6 gene and mtDNA content in SAT that was not observed in visceral adipose tissue. This finding suggests a close relationship between mitochondrial activity and inflammatory response, specifically in SAT [71]. Additionally, mtDNA copy number correlated positively with the expression of CEBPB and CEBPD, indicating a potential influence of mitochondria in regulating adipocyte differentiation and lipid metabolism in SAT [72]. These observations highlight distinct regulatory mechanisms in SAT compared to VAT, suggesting a tissue-specific role of mitochondria in modulating inflammatory and metabolic processes.

In this work, we demonstrated a significant positive correlation between mtDNA copy number and expression of epigenetic regulator genes such as HDAC2, DNMT3a, and DNMT1 in VAT. Epigenetic mechanisms, particularly DNA methylation (regulated by the activity of DNMT1 and DNMT3a) as well as histone deacetylation (regulated mainly by HDAC2), may affect mitochondrial function by modulating the expression of mitochondrial genes [73,74]. These modifications can lead to transcriptional repression or activation of genes encoding proteins involved in mitochondrial metabolism and dynamics, potentially impacting the cellular energy state [43,75]. An increase in mtDNA copy number may suggest enhanced mitochondrial activity in visceral adipose tissue, potentially driven by epigenetic alterations in the regulation of these genes [7]. In the context of VAT, a metabolically active tissue with a central role in lipid storage and energy regulation, such an increase may point to altered metabolic programming potentially driven by epigenetic changes. The modifications in DNMT1, DNMT3A, and HDAC2 may influence the expression of nuclear-encoded mitochondrial-regulatory genes, thereby affecting mitochondrial replication and function [76,77]. Epigenetic regulators may also affect the expression of genes responsible for energy production or lipid metabolism, which may lead to increased mitochondrial number [7]. Visceral adipose tissue is one of the main sites of metabolic dysregulation and inflammation in conditions such as obesity and type 2 diabetes [78]. Epigenetic modifications are known to regulate inflammatory responses, which in turn influence mitochondrial activity and thus mtDNA copy number [79]. Epigenetic factors are primary mechanisms for modulating gene expression related to energy metabolism, and their dysregulation has been linked to metabolic diseases [80]. If these epigenetic regulators are involved in mitochondrial processes, their modulation may impact lipid metabolism and cellular energetics in the context of obesity, insulin resistance, and other metabolic disorders [81]. The observed association between mtDNA copy number and epigenetic regulators in VAT suggests a potential integrative mechanism in which epigenetic reprogramming contributes to mitochondrial adaptation Recent studies have identified alterations in mtDNA methylation, particularly in the D-loop region, which correlated with both mtDNA copy number and the expression of epigenetic enzymes such as DNMTs and HDACs in the context of obesity and metabolic dysfunctions [7,81]. Conversely, the lack of significant correlation between mtDNA copy number and epigenetic regulators in SAT may indicate lower metabolic and inflammatory activity of this tissue compared to VAT. These findings suggest that epigenetic regulation of mitochondrial function may be tissue specific. Therefore, it may be more active in VAT, where they are more closely associated with metabolic disease phenotypes [24,25].

From the perspective of epigenetic regulation, there are many reports indicating that DNMT1, DNMT3A, and HDAC2 modulate the expression of genes related to mitochondrial function. DNMT1 has been shown to methylate and repress the promoters of genes such as PPARα and ADIPOQ in adipocytes, leading to impaired lipid oxidation and insulin sensitivity [82]. DNMT3A, in turn, can suppress expression of FGF21, a key regulator of mitochondrial function and lipid metabolism in adipose tissue by methylation of its promoter region in adipocytes [83]. Meanwhile, HDAC2, as part of class I HDACs, can reduce histone acetylation and PGC-1α expression, which influences mitochondrial biogenesis in adipose tissue [84]. Thus, the correlations observed in our study may reflect a coordinated epigenetic control of genes crucial for mitochondrial content and function, including PGC-1α, PPARα, and others, mediated by DNMT1, DNMT3A, and HDAC2. These findings underscore the relevance of epigenetic mechanisms in shaping mitochondrial phenotypes in VAT, especially under insulin-resistant conditions.

Overall, the presented results show the significant role of mitochondria in visceral adipose tissue in relation to insulin resistance, lipid metabolism, and the insulin signaling pathway. Further large-scale studies are necessary to validate these findings and deepen our understanding of the mitochondrial contribution to adipose tissue function and metabolic disease.

## 4. Materials and Methods

The study protocol was approved by the Ethics Committee Board of Wroclaw Medical University, Approval No. KB-124/2017. The investigations were carried out following the rules of the Declaration of Helsinki.

### 4.1. Study Cohort Inclusion Criteria and Adipose Tissues Collection

Two types of adipose tissue biopsies, subcutaneous (SAT) and visceral (VAT), were collected during abdominal surgery from 27 patients between 2019 and 2021. Patients were recruited to the study based on the following inclusion criteria: (1) age 40–60 years, (2) lack of active cancer disease, (3) no symptoms of chronic inflammation, (4) excluded thyroid dysfunction, and other condition related to IR (eg., PCOS and Cushing Disease), and (5) without alcohol abuse present and past. The study cohort was divided into two study groups, (1) insulin-sensitive (IS), and (2) insulin-resistant (IR), based on the ratios of the HOMA-IR and QUICKI index using the following formulas [85].

(1) HOMA-IR = [(glucose [mmol/l] × insulin [μU/mL])/22.5]
(2) QUICKI = [1/(log glucose [mg/dl] + log insulin [μU/mL])]

Immediately after biopsy collection, the adipose tissue was placed in cold PBS (IITD, Wrocław, Poland) supplemented with protease inhibitor mix (PI, 200×, BioShop, Burlington, ON, Canada) and transported to a laboratory unit. Upon arrival, adipose tissue biopsies were placed in RNALater (Invitrogen) and incubated at 4 °C for 24 h. Then, RNALater was discarded, and tissues were stored at −80 °C until analysis.

Blood was collected before abdominal surgery in a fasted state and transported to the laboratory. The plasma was collected by centrifugation at 4000 rpm for 5 min and frozen at −80 °C until analysis.

The glucose levels, TG, LDL, HDL, and cholesterol were measured in a medical diagnostic laboratory, as part of a clinical procedure using standardized laboratory equipment and reagents CE-IVD approved.

### 4.2. Genetic Material Extraction

The adipose tissues were first homogenized using the MagNa Lyser (Roche, Mannheim, Germany) directly either in TEN buffer (0.2 mM Tris-Cl, pH 7.5, 0.3 mM EDTA, 1 M NaCl, 2% SDS) or in TriReagent (Sigma-Aldrich, St. Louis, MO, USA), for DNA and RNA extraction, respectively. After homogenization, adipose tissue was centrifuged at maximal speed for 5 min at 4 °C. The triglycerides collected at the top of the sample were removed, and the sample proceeded to the next step depending on the type of nucleic acid extraction.

The sample undergoing DNA extraction was digested in TEN buffer with the addition of 50 ng of Proteinase K (Qiagen, Valencia, CA, USA). Next, the phenol/chloroform/isoamyl (25:24:1, BioShop, Burlington, ON, Canada) was added. DNA was precipitated using ethanol (99.8%, Sigma-Aldrich). After washing with 70% ethanol, DNA was suspended in nuclease-free water and stored at −20 °C.

RNA was extracted using the Trizol (Sigma-Aldrich, St. Louis, MO, USA) reagent, followed by chloroform extraction and isopropanol (Sigma-Aldrich, St. Louis, MO, USA) precipitation. Next, RNA was washed with 70% ethanol and dried in air. At the final stage, RNA was suspended in nuclease-free water and stored at −80 °C.

### 4.3. Insulin Level Determination

The Human Insulin ELISA Kit (Sigma-Aldrich) was used for insulin quantification in plasma collected from enrolled individuals according to the manufacturer’s protocol. After signal development, absorbance was read using the Victor3 1420 Multilabel Counter. Insulin concentration was assessed based on the standard curve approach.

### 4.4. Gene Expression

The RNA (200 ng) was transcribed into cDNA using the High-Capacity Reverse Transcription Kit (ThermoFisher, Waltham, MA, USA). Gene expression was analyzed in Real-Time PCR using the Fast SYBR Green Master Mix (ThermoFisher, Waltham, MA, USA) with the use of the 7900HT Fast Real-Time PCR System. Primers were designed manually to span the exon-exon junction. The efficiency of primers was checked by a standard curve; only primers with efficiency 100 ± 5% were used for quantitative analysis. The sequences of primers used for gene expression analysis were published previously [86,87]. The expression was measured as reference Real-Time PCR, results were normalized to the housekeeping gene (β-actin) and calculated according to the ΔΔCt algorithm. Prior to gene expression analysis, the selection of a housekeeping gene was performed, and it was proven that expression of β-actin is not affected by the IR stage.

### 4.5. MtDNA Copy Number Determination

Mitochondrial DNA copy number was determined using DNA extracted from adipose tissue biopsies. Quantification was assessed using the Absolute Human Mitochondrial DNA Copy Number Quantification qPCR Assay Kit (ScienCell™ Research Laboratories, Carlsbad, CA, USA). The quantification of mtDNA was normalized to genomic DNA. Laboratory procedures and quantifications were performed according to the instructions provided by the manufacturer based on Real-Time PCR using the reference standard of known copy numbers of human genomic DNA and mtDNA per diploid cell (724 copies of mtDNA ± 16 per diploid cell). The mtDNA copy number in the analyzed sample was calculated using the ∆∆Cq formula based on Cq values in reference to gDNA. Next, the mtDNA was quantified by normalization to a reference standard using the following formulas:

(1) ∆∆Cq = ∆Cq (mtDNA) − ∆Cq (SCR),

where SCR represents primers specific for gDNA.

(2) mtDNA copy number = (724 ± 16) × 2 − ∆∆Cq,

where (724 ± 16) is the standard concentration

### 4.6. Statistical Analysis

Statistical analysis was performed using Statistica 13.1 (StatSoft). All values are presented as the average ± standard deviation (SD). Before test selection, the normality of distribution was analyzed using Shapiro–Wilk test. For analysis of differences between groups, one-way ANOVA was used. To assess the correlation between numerical characteristics, the correlation coefficient was used. The gene expression was calculated using the ΔΔCt algorithm. The statistical significance was set at *p* < 0.05.

## 5. Conclusions

Our results show that subcutaneous and visceral adipose tissue exhibit distinct metabolic profiles, especially in the context of insulin resistance. VAT, in contrast to SAT, displays a significantly lower mitochondrial DNA (mtDNA) copy number, suggesting reduced mitochondrial activity and a greater susceptibility to inflammation. Insulin resistance in VAT is closely associated with a decrease in mtDNA copy number, indicating mitochondrial dysfunction as a contributing factor in disease progression. Moreover, VAT, in contrast to SAT, exhibits strong correlations between mtDNA copy number and expression of genes involved in insulin signalling, lipid metabolism, and epigenetic regulation, which was not observed in SAT. Our findings highlight the central role of mitochondria in VAT in the context of metabolic disorders and suggest that targeting mitochondrial regulation in this tissue could offer a promising therapeutic strategy.

## Figures and Tables

**Table 1:** Characteristics of studied cohort.

Parameter	IS ^a^ [Mean ± SD]	IR ^b^ [Mean ± SD]	*p* Value
Number [F/M]	13 [4/9]	14 [3/11]	n.s. ^c^
Age [years]	44.2 ± 8	47.7 ± 8	n.s.
BMI [kg/m^2^]	24.6 ± 5.0	27.7 ± 5.1	n.s.
Glucose [mg/dL]	89.9 ± 7	97.7 ± 16	n.s.
Insulin [µU/mL]	7.8 ± 2.7	18.5 ± 13	0.010
HOMA-IR	1.71 ± 0.6	4.3 ± 2.9	0.006
QUICKI	0.359 ± 0.03	0.320 ± 0.03	0.007
Cholesterol [mg/dL]	244 ± 75	200 ± 40	n.s.
TG [mg/dL]	69 ± 22	140 ± 44	<0.0001
LDL [mg/dL]	138 ± 76	128 ± 34	n.s.
HDL [mg/dL]	84 ± 14	45 ± 7	0.005

^a^ IS—insulin-sensitive, ^b^ IR—insulin-resistant, **^c^** n.s.—not statistically significant.

**Table 2:** Statistical characterization of mtDNA content in SAT and VAT of enrolled subjects.

mtDNA	IS [Mean ± SD]	IR [Mean ± SD]	*p* Value
SAT	21.736 ± 39.812	4089 ± 4162	0.159
VAT	11.471 ± 13.790	2288 ± 1004	0.031

**Table 3:** Correlation between mtDNA copy number in SAT and VAT and clinical characteristics.

Parameter	SAT		VAT	
	R^2^	*p* Value	R^2^	*p* Value
Age [years]	−0.62	0.002	−0.05	n.s. ^a^
BMI [kg/m^2^]	−0.17	n.s.	−0.57	0.050
Glucose [mg/dL]	−0.17	n.s.	−0.07	n.s.
Insulin [µU/mL]	0.04	n.s.	−0.31	n.s.
HOMA-IR	0.00	n.s.	−0.31	n.s.
QUICKI	−0.05	n.s.	0.51	0.014
Cholesterol [mg/dL]	−0.07	n.s.	0.05	n.s.
TG [mg/dL]	−0.22	n.s.	−0.28	n.s.
LDL [mg/dL]	0.25	n.s.	0.32	n.s.
HDL [mg/dL]	−0.11	n.s.	0.02	n.s.

^a^ n.s.—statistically not significant.

**Table 4:** The correlation between the expression of numerous genes measured in VAT and SAT with mtDNA copy number detected in particular fat depots of the analyzed subjects.

Gene	SAT				VAT			
	IR		IS		IR		IS	
	R^2^	*p* Value	R^2^	*p* Value	R^2^	*p* Value	R^2^	*p* Value
*HDAC1*	−0.13	n.s.	−0.09	n.s.	0.05	n.s.	0.60	0.043
*HDAC2*	−0.08	n.s.	−0.19	n.s.	0.66	0.021	0.84	0.008
*SIRT 1*	−0.10	n.s.	−0.12	n.s.	0.83	0.001	0.56	n.s.
*INSR*	−0.37	n.s.	−0.28	n.s.	0.77	0.003	0.47	n.s.
*AKT*	−0.16	n.s.	−0.31	n.s.	0.69	0.015	0.08	n.s.
*SLC2A4*	−0.42	n.s.	0.04	n.s.	0.91	<0.0001	0.73	0.008
*PPARG*	−0.26	n.s.	−0.06	n.s.	0.87	<0.0001	0.24	n.s.
*IL6*	0.28	n.s.	0.99	<0.0001	−0.27	n.s.	−0.23	n.s.
*IL10*	0.17	n.s.	−0.08	n.s.	−0.22	n.s.	−0.17	n.s.
*PIK3R1*	−0.11	n.s.	−0.05	n.s.	0.88	0.001	0.61	0.036
*LEP*	−0.36	n.s.	−0.32	n.s.	0.03	n.s.	−0.28	n.s.
*ADIPOQ*	−0.41	n.s.	−0.14	n.s.	0.84	<0.0001	−0.15	n.s.
*RBP4*	−0.35	n.s.	−0.14	n.s.	0.78	0.003	−0.02	n.s.
*LPL*	−0.38	n.s.	−0.19	n.s.	0.78	0.003	0.08	n.s.
*ACC*	−0.57	n.s.	−0.16	n.s.	0.88	<0.0001	0.28	n.s.
*DNMT1*	0.31	n.s.	0.12	n.s.	0.71	0.011	0.57	n.s.
*DNMT3A*	−0.15	n.s.	−0.11	n.s.	0.38	n.s.	0.92	0.001
*CEBPA*	−0.13	n.s.	−0.01	n.s.	0.80	0.002	−0.26	n.s.
*CEBPB*	0.96	0.008	0.97	0.012	0.44	n.s.	0.03	n.s.
*CEPBD*	0.96	0.001	0.99	0.001	−0.04	n.s.	−0.10	n.s.
*TNF*	0.03	n.s.	0.11	n.s.	−0.21	n.s.	−0.02	n.s.
*FASN*	−0.45	n.s.	−0.15	n.s.	0.91	<0.0001	0.32	n.s.
*ACSS2*	−0.41	n.s.	0.11	n.s.	0.87	0.003	0.79	0.002
*SCD1*	−0.28	n.s.	−0.13	n.s.	0.59	0.046	−0.05	n.s.
*SIRT7*	0.31	n.s.	−0.01	n.s.	0.58	0.049	0.21	n.s.
*HIF3a*	0.42	n.s.	−0.09	n.s.	0.06	n.s.	−0.07	n.s.
*PGC1a*	−0.38	n.s.	−0.28	n.s.	−0.22	n.s.	0.82	0.001
*IGF2*	0.23	n.s.	0.16	n.s.	−0.04	n.s.	−0.09	n.s.

n.s.—statistically not significant.

## Data Availability

The raw data supporting the conclusion of this article will be shared upon request to the corresponding author.

## Abbreviations

The following abbreviations are used in this manuscript:

IR	insulin-resistant
IS	insulin-sensitive
mtDNA	mitochondrial DNA
ROS	reactive oxygen species
SAT	subcutaneous adipose tissue
VAT	visceral adipose tissue

## References

[1] Lee S.-H., Park S.-Y., Choi C.S. (2021). Insulin Resistance: From Mechanisms to Therapeutic Strategies. Diabetes Metab. J..

[2] Kim J., Wei Y., Sowers J.R. (2008). Role of Mitochondrial Dysfunction in Insulin Resistance. Circ. Res..

[3] Brand M.D., Orr A.L., Perevoshchikova I.V., Quinlan C.L. (2013). The Role of Mitochondrial Function and Cellular Bioenergetics in Ageing and Disease. Br. J. Dermatol..

[4] Asmann Y.W., Stump C.S., Short K.R., Coenen-Schimke J.M., Guo Z., Bigelow M.L., Nair K.S. (2006). Skeletal Muscle Mitochondrial Functions, Mitochondrial DNA Copy Numbers, and Gene Transcript Profiles in Type 2 Diabetic and Nondiabetic Subjects at Equal Levels of Low or High Insulin and Euglycemia. Diabetes.

[5] Petersen M.C., Shulman G.I. (2018). Mechanisms of Insulin Action and Insulin Resistance. Physiol. Rev..

[6] Stump C.S., Short K.R., Bigelow M.L., Schimke J.M., Nair K.S. (2003). Effect of Insulin on Human Skeletal Muscle Mitochondrial ATP Production, Protein Synthesis, and mRNA Transcripts. Proc. Natl. Acad. Sci. USA.

[7] Bordoni L., Perugini J., Petracci I., Mercurio E.D., Lezoche G., Guerrieri M., Giordano A., Gabbianelli R. (2022). Mitochondrial DNA in Visceral Adipose Tissue in Severe Obesity: From Copy Number to D-Loop Methylation. Front. Biosci. Landmark.

[8] DeBarmore B., Longchamps R.J., Zhang Y., Kalyani R.R., Guallar E., Arking D.E., Selvin E., Young J.H. (2020). Mitochondrial DNA Copy Number and Diabetes: The Atherosclerosis Risk in Communities (ARIC) Study. BMJ Open Diabetes Res. Care.

[9] Todosenko N., Khaziakhmatova O., Malashchenko V., Yurova K., Bograya M., Beletskaya M., Vulf M., Gazatova N., Litvinova L. (2023). Mitochondrial Dysfunction Associated with mtDNA in Metabolic Syndrome and Obesity. Int. J. Mol. Sci..

[10] Szendroedi J., Phielix E., Roden M. (2011). The Role of Mitochondria in Insulin Resistance and Type 2 Diabetes Mellitus. Nat. Rev. Endocrinol..

[11] Sangwung P., Petersen K.F., Shulman G.I., Knowles J.W. (2020). Mitochondrial Dysfunction, Insulin Resistance, and Potential Genetic Implications. Endocrinology.

[12] Pagel-Langenickel I., Bao J., Pang L., Sack M.N. (2010). The Role of Mitochondria in the Pathophysiology of Skeletal Muscle Insulin Resistance. Endocr. Rev..

[13] Sultana M.A., Hia R.A., Akinsiku O., Hegde V. (2023). Peripheral Mitochondrial Dysfunction: A Potential Contributor to the Development of Metabolic Disorders and Alzheimer’s Disease. Biology.

[14] Bhatti J.S., Bhatti G.K., Reddy P.H. (2017). Mitochondrial Dysfunction and Oxidative Stress in Metabolic Disorders—A Step towards Mitochondria Based Therapeutic Strategies. Biochim. Biophys. Acta.

[15] Song J., Oh J.Y., Sung Y.-A., Pak Y.K., Park K.S., Lee H.K. (2001). Peripheral Blood Mitochondrial DNA Content Is Related to Insulin Sensitivity in Offspring of Type 2 Diabetic Patients. Diabetes Care.

[16] Mootha V.K., Lindgren C.M., Eriksson K.-F., Subramanian A., Sihag S., Lehar J., Puigserver P., Carlsson E., Ridderstråle M., Laurila E. (2003). PGC-1α-Responsive Genes Involved in Oxidative Phosphorylation Are Coordinately Downregulated in Human Diabetes. Nat. Genet..

[17] Petersen K.F., Dufour S., Befroy D., Garcia R., Shulman G.I. (2004). Impaired Mitochondrial Activity in the Insulin-Resistant Offspring of Patients with Type 2 Diabetes. N. Engl. J. Med..

[18] Boudina S., Graham T.E. (2014). Mitochondrial Function/Dysfunction in White Adipose Tissue. Exp. Physiol..

[19] Lee J.H., Park A., Oh K.-J., Lee S.C., Kim W.K., Bae K.-H. (2019). The Role of Adipose Tissue Mitochondria: Regulation of Mitochondrial Function for the Treatment of Metabolic Diseases. Int. J. Mol. Sci..

[20] Kusminski C.M., Scherer P.E. (2012). Mitochondrial Dysfunction in White Adipose Tissue. Trends Endocrinol. Metab. TEM.

[21] Montgomery M.K., Turner N. (2014). Mitochondrial Dysfunction and Insulin Resistance: An Update. Endocr. Connect..

[22] He F., Huang Y., Song Z., Zhou H.J., Zhang H., Perry R.J., Shulman G.I., Min W. (2021). Mitophagy-Mediated Adipose Inflammation Contributes to Type 2 Diabetes with Hepatic Insulin Resistance. J. Exp. Med..

[23] Vernochet C., Damilano F., Mourier A., Bezy O., Mori M.A., Smyth G., Rosenzweig A., Larsson N.-G., Kahn C.R. (2014). Adipose Tissue Mitochondrial Dysfunction Triggers a Lipodystrophic Syndrome with Insulin Resistance, Hepatosteatosis, and Cardiovascular Complications. FASEB J..

[24] Ibrahim M.M. (2010). Subcutaneous and Visceral Adipose Tissue: Structural and Functional Differences. Obes. Rev..

[25] Wajchenberg B.L. (2000). Subcutaneous and Visceral Adipose Tissue: Their Relation to the Metabolic Syndrome. Endocr. Rev..

[26] Shulman G.I. (2014). Ectopic Fat in Insulin Resistance, Dyslipidemia, and Cardiometabolic Disease. N. Engl. J. Med..

[27] Kraunsøe R., Boushel R., Hansen C.N., Schjerling P., Qvortrup K., Støckel M., Mikines K.J., Dela F. (2010). Mitochondrial Respiration in Subcutaneous and Visceral Adipose Tissue from Patients with Morbid Obesity. J. Physiol..

[28] Takano C., Ogawa E., Hayakawa S. (2023). Insulin Resistance in Mitochondrial Diabetes. Biomolecules.

[29] Palikaras K., Lionaki E., Tavernarakis N. (2018). Mechanisms of mitophagy in cellular homeostasis, physiology and pathology. Nat. Cell Biol..

[30] Kurihara Y., Kanki T., Aoki Y., Hirota Y., Saigusa T., Uchiumi T., Kang D. (2012). Mitophagy Plays an Essential Role in Reducing Mitochondrial Production of Reactive Oxygen Species and Mutation of Mitochondrial DNA by Maintaining Mitochondrial Quantity and Quality in Yeast. J. Biol. Chem..

[31] Amaral A., Ramalho-Santos J., John J.C.S. (2007). The expression of polymerase gamma and mitochondrial transcription factor A and the regulation of mitochondrial DNA content in mature human sperm. Hum. Reprod..

[32] Gorman G.S., Chinnery P.F., DiMauro S., Hirano M., Koga Y., McFarland R., Suomalainen A., Thorburn D.R., Zeviani M., Turnbull D.M. (2016). Mitochondrial diseases. Nat. Rev. Dis. Prim..

[33] Ekstrand M.I., Falkenberg M., Rantanen A., Park C.B., Gaspari M., Hultenby K., Rustin P., Gustafsson C.M., Larsson N.-G. (2004). Mitochondrial transcription factor A regulates mtDNA copy number in mammals. Hum. Mol. Genet..

[34] Stiles A.R., Simon M.T., Stover A., Eftekharian S., Khanlou N., Wang H.L., Magaki S., Lee H., Partynski K., Dorrani N. (2016). Mutations in TFAM, encoding mitochondrial transcription factor A, cause neonatal liver failure associated with mtDNA depletion. Mol. Genet. Metab..

[35] Stewart J.D., Schoeler S., Sitarz K.S., Horvath R., Hallmann K., Pyle A., Yu-Wai-Man P., Taylor R.W., Samuels D.C., Kunz W.S. (2011). POLG mutations cause decreased mitochondrial DNA repopulation rates following induced depletion in human fibroblasts. Biochim. et Biophys. Acta (BBA). Mol. Basis Dis..

[36] Lee H. (2004). Somatic mutations in the D-loop and decrease in the copy number of mitochondrial DNA in human hepatocellular carcinoma. Mutat. Res. Mol. Mech. Mutagen..

[37] Kong M., Guo L., Xu W., He C., Jia X., Zhao Z., Gu Z. (2022). Aging-associated accumulation of mitochondrial DNA mutations in tumor origin. Life Med..

[38] Liao S., Chen L., Song Z., He H. (2022). The Fate of Damaged Mitochondrial DNA in the Cell. Biochim. Biophys. Acta BBA Mol. Cell Res..

[39] Abd Radzak S.M., Mohd Khair S.Z.N., Ahmad F., Patar A., Idris Z., Mohamed Yusoff A.A. (2022). Insights Regarding Mitochondrial DNA Copy Number Alterations in Human Cancer (Review). Int. J. Mol. Med..

[40] Lee J.-Y., Lee D.-C., Im J.-A., Lee J.-W. (2014). Mitochondrial DNA Copy Number in Peripheral Blood Is Independently Associated with Visceral Fat Accumulation in Healthy Young Adults. Int. J. Endocrinol..

[41] Sergi D., Naumovski N., Heilbronn L.K., Abeywardena M., O’Callaghan N., Lionetti L., Luscombe-Marsh N. (2019). Mitochondrial (Dys)Function and Insulin Resistance: From Pathophysiological Molecular Mechanisms to the Impact of Diet. Front. Physiol..

[42] Zorzano A., Liesa M., Palacín M. (2009). Mitochondrial Dynamics as a Bridge between Mitochondrial Dysfunction and Insulin Resistance. Arch. Physiol. Biochem..

[43] Scarpulla R.C., Vega R.B., Kelly D.P. (2012). Transcriptional Integration of Mitochondrial Biogenesis. Trends Endocrinol. Metab. TEM.

[44] Boucher J., Kleinridders A., Kahn C.R. (2014). Insulin Receptor Signaling in Normal and Insulin-Resistant States. Cold Spring Harb. Perspect. Biol..

[45] Zhou Q., Yu J., Yuan X., Wang C., Zhu Z., Zhang A., Gu W. (2021). Clinical and Functional Characterization of Novel INSR Variants in Two Families With Severe Insulin Resistance Syndrome. Front. Endocrinol..

[46] Gao Z., Hwang D., Bataille F., Lefevre M., York D., Quon M.J., Ye J. (2002). Serine Phosphorylation of Insulin Receptor Substrate 1 by Inhibitor Kappa B Kinase Complex. J. Biol. Chem..

[47] Guilherme A., Virbasius J.V., Puri V., Czech M.P. (2008). Adipocyte Dysfunctions Linking Obesity to Insulin Resistance and Type 2 Diabetes. Nat. Rev. Mol. Cell Biol..

[48] Morigny P., Houssier M., Mouisel E., Langin D. (2016). Adipocyte Lipolysis and Insulin Resistance. Biochimie.

[49] Christen T., Sheikine Y., Rocha V.Z., Hurwitz S., Goldfine A.B., Di Carli M., Libby P. (2010). Increased Glucose Uptake in Visceral Versus Subcutaneous Adipose Tissue Revealed by PET Imaging. JACC Cardiovasc. Imaging.

[50] Kojta I., Chacińska M., Błachnio-Zabielska A. (2020). Obesity, Bioactive Lipids, and Adipose Tissue Inflammation in Insulin Resistance. Nutrients.

[51] Jin X., Qiu T., Li L., Yu R., Chen X., Li C., Proud C.G., Jiang T. (2023). Pathophysiology of Obesity and Its Associated Diseases. Acta Pharm. Sin. B.

[52] Wróblewski A., Strycharz J., Oszajca K., Czarny P., Świderska E., Matyjas T., Zieleniak A., Rucińska M., Pomorski L., Drzewoski J. (2023). Dysregulation of Inflammation, Oxidative Stress, and Glucose Metabolism-Related Genes and miRNAs in Visceral Adipose Tissue of Women with Type 2 Diabetes Mellitus. Med. Sci. Monit. Int. Med. J. Exp. Clin. Res..

[53] Rutanen J., Yaluri N., Modi S., Pihlajamäki J., Vänttinen M., Itkonen P., Kainulainen S., Yamamoto H., Lagouge M., Sinclair D.A. (2010). SIRT1 mRNA Expression May Be Associated With Energy Expenditure and Insulin Sensitivity. Diabetes.

[54] Walczak R., Tontonoz P. (2002). PPARadigms and PPARadoxes: Expanding Roles for PPARγ in the Control of Lipid Metabolism. J. Lipid Res..

[55] Rosen E.D., Hsu C.-H., Wang X., Sakai S., Freeman M.W., Gonzalez F.J., Spiegelman B.M. (2002). C/EBPα Induces Adipogenesis through PPARγ: A Unified Pathway. Genes Dev..

[56] Ziegler A.K., Scheele C. (2024). Human Adipose Depots’ Diverse Functions and Dysregulations during Cardiometabolic Disease. Npj Metab. Health Dis..

[57] Dhokte S., Czaja K. (2024). Visceral Adipose Tissue: The Hidden Culprit for Type 2 Diabetes. Nutrients.

[58] Gabriely I., Ma X.H., Yang X.M., Atzmon G., Rajala M.W., Berg A.H., Scherer P., Rossetti L., Barzilai N. (2002). Removal of Visceral Fat Prevents Insulin Resistance and Glucose Intolerance of Aging: An Adipokine-Mediated Process?. Diabetes.

[59] Boone C., Lewis S.C. (2024). Bridging Lipid Metabolism and Mitochondrial Genome Maintenance. J. Biol. Chem..

[60] Villarroya J., Giralt M., Villarroya F. (2009). Mitochondrial DNA: An Up-and-Coming Actor in White Adipose Tissue Pathophysiology. Obesity.

[61] Kaaman M., Sparks L.M., van Harmelen V., Smith S.R., Sjölin E., Dahlman I., Arner P. (2007). Strong Association between Mitochondrial DNA Copy Number and Lipogenesis in Human White Adipose Tissue. Diabetologia.

[62] Scarpulla R.C. (2006). Nuclear Control of Respiratory Gene Expression in Mammalian Cells. J. Cell. Biochem..

[63] Cecil J.E., Watt P., Palmer C.N., Hetherington M. (2006). Energy Balance and Food Intake: The Role of PPARgamma Gene Polymorphisms. Physiol. Behav..

[64] Li X. (2013). SIRT1 and Energy Metabolism. Acta Biochim. Biophys. Sin..

[65] Liang H., Ward W.F. (2006). PGC-1α: A Key Regulator of Energy Metabolism. Adv. Physiol. Educ..

[66] Cedikova M., Kripnerová M., Dvorakova J., Pitule P., Grundmanova M., Babuska V., Mullerova D., Kuncova J. (2016). Mitochondria in White, Brown, and Beige Adipocytes. Stem Cells Int..

[67] Van Nguyen T.-T., Van Vu V., Van Pham P. (2023). Brown Adipocyte and Browning Thermogenesis: Metabolic Crosstalk beyond Mitochondrial Limits and Physiological Impacts. Adipocyte.

[68] Deng T., Sieglaff D.H., Zhang A., Lyon C.J., Ayers S.D., Cvoro A., Gupte A.A., Xia X., Baxter J.D., Webb P. (2011). A Peroxisome Proliferator-Activated Receptor γ (PPARγ)/PPARγ Coactivator 1β Autoregulatory Loop in Adipocyte Mitochondrial Function. J. Biol. Chem..

[69] Lefterova M.I., Lazar M.A. (2009). New Developments in Adipogenesis. Trends Endocrinol. Metab..

[70] Guerrier L., Malpuech-Brugère C., Richard R., Touron J. (2023). Mitochondrial Function in Healthy Human White Adipose Tissue: A Narrative Review. Nutrients.

[71] Petersen K.F., Shulman G.I. (2006). Etiology of Insulin Resistance. Am. J. Med..

[72] Rosen E.D., MacDougald O.A. (2006). Adipocyte Differentiation from the inside Out. Nat. Rev. Mol. Cell Biol..

[73] Kwon D.-H., Ryu J., Kim Y.-K., Kook H. (2020). Roles of Histone Acetylation Modifiers and Other Epigenetic Regulators in Vascular Calcification. Int. J. Mol. Sci..

[74] Maresca A., Zaffagnini M., Caporali L., Carelli V., Zanna C. (2015). DNA Methyltransferase 1 Mutations and Mitochondrial Pathology: Is mtDNA Methylated?. Front. Genet..

[75] Wallace D.C., Fan W. (2010). Energetics, Epigenetics, Mitochondrial Genetics. Mitochondrion.

[76] Devall M., Roubroeks J., Mill J., Weedon M., Lunnon K. (2016). Epigenetic Regulation of Mitochondrial Function in Neurodegenerative Disease: New Insights from Advances in Genomic Technologies. Neurosci. Lett..

[77] Wellen K.E., Hotamisligil G.S. (2005). Inflammation, Stress, and Diabetes. J. Clin. Investig..

[78] Makki K., Froguel P., Wolowczuk I. (2013). Adipose Tissue in Obesity-Related Inflammation and Insulin Resistance: Cells, Cytokines, and Chemokines. Int. Sch. Res. Not..

[79] Chatterjee D., Das P., Chakrabarti O. (2022). Mitochondrial Epigenetics Regulating Inflammation in Cancer and Aging. Front. Cell Dev. Biol..

[80] Gao W., Liu J.-L., Lu X., Yang Q. (2021). Epigenetic Regulation of Energy Metabolism in Obesity. J. Mol. Cell Biol..

[81] Zheng L.D., Linarelli L.E., Liu L., Wall S.S., Greenawald M.H., Seidel R.W., Estabrooks P.A., Almeida F.A., Cheng Z. (2015). Insulin Resistance Is Associated with Epigenetic and Genetic Regulation of Mitochondrial DNA in Obese Humans. Clin. Epigenetics.

[82] Kim A.Y., Park Y.J., Pan X., Shin K.C., Kwak S.-H., Bassas A.F., Sallam R.M., Park K.S., Alfadda A.A., Xu A. (2015). Obesity-Induced DNA Hypermethylation of the Adiponectin Gene Mediates Insulin Resistance. Nat. Commun..

[83] You D., Nilsson E., Tenen D.E., Lyubetskaya A., Lo J.C., Jiang R., Deng J., Dawes B.A., Vaag A., Ling C. (2017). Dnmt3a Is an Epigenetic Mediator of Adipose Insulin Resistance. eLlife.

[84] Galmozzi A., Mitro N., Ferrari A., Gers E., Gilardi F., Godio C., Cermenati G., Gualerzi A., Donetti E., Rotili D. (2013). Inhibition of Class I Histone Deacetylases Unveils a Mitochondrial Signature and Enhances Oxidative Metabolism in Skeletal Muscle and Adipose Tissue. Diabetes.

[85] Ruano M., Silvestre V., Castro R., García-Lescún M.C.G., Aguirregoicoa E., Marco A., Rodríguez A., García-Blanch G. (2006). HOMA, QUICKI and MFfm to Measure Insulin Resistance in Morbid Obesity. Obes. Surg..

[86] Cierzniak A., Pawelka D., Kaliszewski K., Rudnicki J., Dobosz T., Malodobra-Mazur M. (2021). DNA methylation in adipocytes from visceral and subcutaneous adipose tissue influences insulin-signaling gene expression in obese individuals. Int. J. Obes..

[87] Małodobra-Mazur M., Cierzniak A., Pawełka D., Kaliszewski K., Rudnicki J., Dobosz T. (2020). Metabolic Differences between Subcutaneous and Visceral Adipocytes Differentiated with an Excess of Saturated and Monounsaturated Fatty Acids. Genes.

